# Recent Chikungunya Virus Infection in 2 Travelers Returning from Mogadishu, Somalia, to Italy, 2016

**DOI:** 10.3201/eid2211.161225

**Published:** 2016-11

**Authors:** Lorenzo Zammarchi, Claudia Fortuna, Giulietta Venturi, Francesca Rinaldi, Teresa Capobianco, Maria Elena Remoli, Gian Maria Rossolini, Giovanni Rezza, Alessandro Bartoloni

**Affiliations:** Università Degli Studi di Firenze, Florence, Italy (L. Zammarchi, F. Rinaldi, G.M. Rossolini, A. Bartoloni);; Azienda Ospedaliero-Universitaria Careggi, Florence (L. Zammarchi, T. Capobianco, G.M. Rossolini, A. Bartoloni);; Istituto Superiore di Sanità, Rome, Italy (C. Fortuna, G. Venturi, M.E. Remoli, G. Rezza);; Università di Siena, Siena, Italy (G.M. Rossolini);; Fondazione IRCCS Don Carlo Gnocchi, Florence (G.M. Rossolini)

**Keywords:** Chikungunya virus, Italy, Europe, Somalia, Africa, traveler, viruses

**To the Editor:** Since chikungunya virus (CHIKV) was first isolated in 1952 (in Tanzania), outbreaks have occurred every 7–20 years in countries in Africa and Asia, and since 2013, it has been identified in the Americas (*1*,*2*). However, no cases have been reported from the Horn of Africa (*3*,*4*). We confirmed CHIKV infection acquired in 2016 by 2 travelers to Somalia who returned to Italy.

In June 2016, a Somali woman (patient 1) was referred to the Infectious and Tropical Diseases Unit, Careggi University Hospital, in Florence, Italy, because of severe diffuse bilateral arthralgia and edema in hands, wrists, ankles, and feet. Five days earlier, she had returned to Italy from Mogadishu, Somalia, where she had spent 45 days visiting relatives. The woman had been living in Italy since the 1990s and returned to Somalia each year for ≈2 months; she denied travel to other countries. She reported that symptoms started abruptly in May, 17 days after arriving in Somalia (28 days before returning to Italy). At symptom onset, arthralgia was associated with fever and skin rash, which lasted a few days.

In early July 2016, another Somali woman (patient 2) with bilateral arthralgia in her hands, wrists, ankles, and feet associated with foot edema sought medical care at the same hospital 7 days after returning from a 65-day trip to Mogadishu, where she visited relatives. The woman had been living in Italy ≈20 years; the only other travel she reported was to Kenya in 2012. Her symptoms started in June, 20 days after arriving in Somalia (45 days before returning to Italy). At symptom onset, she also had skin rash and fever, which lasted a few days.

Both patients reported that, during the same period, some of their relatives in Mogadishu had similar symptoms and were clinically diagnosed as having chikungunya fever by local doctors. Both also reported that, during the same period, other cases had been reported in Mogadishu by mass and social media and, thus, the local population was aware of the disease.

Serum samples for patients 1 and 2 were positive for CHIKV antibodies (Table). Both patients were treated with nonsteroidal antiinflammatory drugs and corticosteroids and are receiving follow-up.

**Table:** Results of chikungunya virus testing for 2 persons who returned to Italy after traveling to Mogadishu, Somalia, 2016*

Laboratory test performed	Place where test was performed	Results	
		Patient 1†	Patient 2‡
OnSite Chikungunya IgM Combo Rapid Test-Cassette (CTK Biotech, San Diego CA, USA)§	Careggi University Hospital	Positive	Negative
Chikungunya virus IFA IgG (Euroimmun AG, Luebeck, Germany)¶	Careggi University Hospital	Titer >1:100#	Titer >1:100#
Chikungunya virus IFA IgM (Euroimmun)**	Careggi University Hospital	Positive	Positive
Anti-CHIKV IgM ELISA (Euroimmun)††	ISS, National Reference Laboratory for Arboviruses	Index 7.9‡‡	Index 3.4‡‡
PRNT for Chikungunya virus	ISS, National Reference Laboratory for Arboviruses	PRNT80 >1:10§§	PRNT80 >1:10§§

*Testing was conducted on samples taken the day patients 1 and 2 sought care at the Infectious and Tropical Diseases Outpatient Unit at Careggi University Hospital in Florence, Italy. IFA, immunofluorescence assay; ISS, National Institute of Health in Rome, Italy; PRNT, plaque reduction neutralization test; PRNT80, 80% PRNT. †Samples were obtained 33 d after symptom onset.  ‡Samples were obtained 52 d after symptom onset.  §Sensitivity/specificity 90.3%/100% according to information reported in the kit data sheet (reference *10* in Technical Appendix, and 30%/73% according to an independent evaluation (reference *11* in Technical Appendix). ¶Sensitivity/specificity 100%/96% according to information reported in the kit data sheet (reference *12* in Technical Appendix).  #Cut-off for positivity >1:10.  **Sensitivity/specificity 95%/96% according to information reported in the kit data sheet (reference *12* in Technical Appendix).  ††Sensitivity/specificity 98.1%/98.9% according to information reported in the kit data sheet (reference *13* in Technical Appendix) and 85%/82% according to an independent evaluation (reference *11* in Technical Appendix). ‡‡Cut-off for positivity >1.1.  §§PRNT80 titers >1:10 are considered positive.

According to the US Centers for Disease Control and Prevention, as of April 22, 2016, CHIKV had not been reported from Somalia (*4*), and no evidence exists for CHIKV circulation in that area of the Horn of Africa (*3*). In addition, on August 3, 2016, we performed a literature search in PubMed, Embase, and ProMED-mail, and found no reports of CHIKV in Somalia. Poorly documented preliminary data on the presence of CHIKV in Somalia were recently reported in 2 documents by the United Nations Office for the Coordination of Humanitarian Affairs. One document, dated June 7, 2016, stated “There are reports of an outbreak of the deadly Chikungunya virus in Banadir Region. According to WHO [the World Health Organization], 3 of 5 blood samples have tested positive” (reference *1* in Technical Appendix). The second document, dated June 30, 2016, stated that “some 11 suspected cases of Chikungunya were confirmed... in Mogadishu” (reference *2* in Technical Appendix). Several reports in the online press and social media have reported the current circulation of CHIKV in Somalia, including 2 Twitter posts (tweets) by the Ministry of Health of Kenya (references *3–7* in Technical Appendix). A Somali doctor living in Italy obtained confirmation of CHIKV circulation in Somalia by contacting colleagues at the Ministry of Health in Mogadishu (Omar Abdulcadir, Careggi University Hospital, pers. comm., 2016 Jul 19).

Direct and indirect evidence exists for the presence of competent CHIKV vectors (e.g., *Aedes aegypti* mosquitoes) in Somalia. Entomologic studies conducted in 1942 and 1969 confirmed the presence of *A. aegypti* mosquitoes in several cities along Somalia’s coast, including Mogadishu (*5*). Other arboviruses in which *Aedes* mosquitoes play a role as reservoir (Rift valley fever [RVF]) and vector (dengue and possibly RVF) have been reported in recent years in Somalia: RVF outbreaks occurred during 1997–1998 and 2006–2007 (*6*,*7*), and a dengue outbreak occurred during 1992–1993 (*8*).

The current outbreak in Somalia could have been triggered by several factors, including circulation of CHIKV in neighboring Kenya (references *8*,*9* in online Technical Appendix) and heavy rains that led to flooding in southern and central Somalia beginning in January 2016 (reference *1* in Technical Appendix). CHIKV has the potential to provoke explosive outbreaks in naive populations (*9*), so the current outbreak may greatly affect the economy and public health in Somalia.

Systematic studies to understand the magnitude of the ongoing epidemic are needed. In the meantime, local public health stakeholders in Somalia and healthcare workers worldwide caring for travelers returning from Somalia should be aware that CHIKV is circulating in the country. This report confirms the importance of travel medicine services in performing early diagnosis of imported arboviral diseases, not only to thwart secondary transmission during periods of competent vector activity but also to help to detect or confirm virus circulation in previously unaffected countries.

Technical Appendix Additional references.
