# Supplementary material for: Recent Chikungunya Virus Infection in 2 Travelers Returning from Mogadishu, Somalia, to Italy, 2016
Source: Emerg Infect Dis. 2016 Nov;22(11):2025–7. doi: 10.3201/eid2211.161225 (PMC5088032; doi:10.3201/eid2211.161225)
Supplement: Technical Appendix — Additional references. [file 16-1225-Techapp-s1.pdf]

Article DOI: http://dx.doi.org/10.3201/eid2211.161225

Recent Chikungunya Virus Infection in 2 Travelers Returning from Mogadishu, Somalia, to Italy, 2016

Technical Appendix

Additional References

1. United Nations Office for the Coordination of Humanitarian Affairs. Somalia: humanitarian Snapshot (as of 7 June 2016) [cited 2016 Jul 19]. http://reliefweb.int/report/somalia/somalia-humanitarian-snapshot-7-june-2016

2. UN Office for the Coordination of Humanitarian Affairs. Somalia: humanitarian Dashboard–May 2016 (issued on 30 June 2016) [cited 2016 Jul 19]. http://reliefweb.int/sites/reliefweb.int/files/resources/Somalia%20Humanitarian%20Dashboard%20-%20May2016.pdf

3. Nep Journal. The Kuduudiye/Kuduudshe fever (chikungunya fever) [cited 2016 Jul 19] http://nepjournal.com/the-kuduudiyekuduudshe-fever-chikungunya-fever/

4. Muraya J. Alarm over chikungunya outbreak [cited 2016 Jul 19]. http://www.standardmedia.co.ke/health/article/2000203242/alarm-over-chikungunya-outbreak

5. Hage. Kaduudiye oo kurbo ku haya Soomaalida. Jariiban News Network. 2016 Jun 18 [cited 2016 Jul 19]. http://www.jariibannews.com/archives/5162

6. Ministry of Health Kenya. We shall also conduct a cross border meeting with Somalia & Ethiopia on #Chikungunya in order to effectively manage the situation [cited 2016 Jul 19]. https://twitter.com/MOH_Kenya/status/735765173017808897?s=03

7. Ministry of Health Kenya. It is important to coordinate intervention efforts with Somalia & Ethiopia given that Somalia recently experienced a #Chikungunya outbreak [cited 2016 Jul 29]. https://twitter.com/MOH_Kenya/status/737361939261706240?s=03

8. ProMED Mail. Chikungunya (11): Americas, Pacific, Asia, Africa. ProMed. 2016 Jul 2 [cited 2016 Jul 29]. http://www.promedmail.org, archive no. 20160702.4323117.

9. Medecins sans Frontiers. Double outbreak of cholera and chikungunya placing huge strain on Mandera medical services [cited 2016 Jul 19]. https://www.ecoi.net/local_link/324862/451009_en.html

10. Biotech CTK. Inc. OnSite Chikungunya IgM Combo Rapid Test-Cassette. Package insert PI-R0066 Rev. 2007 [cited cited 2016 Jul 19]. http://www.harmony-vos.sk/ORGENICS/PRIBALOVE/RAPID/R0066C-Chikungunya.pdf

11. Prat CM, Flusin O, Panella A, Tenebray B, Lanciotti R, Leparc-Goffart I. Evaluation of commercially available serologic diagnostic tests for chikungunya virus. Emerg Infect Dis. 2014;20:2129–32. PubMed http://dx.doi.org/10.3201/eid2012.141269

12. Euroimmun AG. Chikungunya virus IFA IgG and IgM. Package insert. Version 09/2014 FI_293a_D_UK_A03. 2014 [cited 2016 Jul 19]. https://www.euroimmun.com/documents/Indications/Infections/Ermerging-viruses-and-parasites/FI_293a_D_UK_A.pdf

13. Euroimmun AG. Anti-CHIKV IgM ELISA. Package insert. Version: 17/12/2015 EI_293aM_A_UK_C04. 2015 [cited 2016 Jul 19]. http://www.fishersci.com/content/dam/fishersci/en_US/documents/programs/healthcare/technical-documents/package-inserts/chikungunya-test-package-insert.pdf
